# Supplementary material for: Cost-consequence analysis of ambulatory clinic- and home-based multidrug-resistant tuberculosis management models in Eswatini
Source: PLoS One. 2024 Apr 2;19(4):e0301507. doi: 10.1371/journal.pone.0301507 (PMC10986922; doi:10.1371/journal.pone.0301507)
Supplement: S1 Table — (DOCX) [file pone.0301507.s001.docx]

**S1 Table: Unit costs of components associated with diagnosis and treatment of MDR-TB**

| **Cost component** | **Unit cost** | **Source** |
| --- | --- | --- |
| **I. Laboratory and radiology (per test)** |  | Pooran et al.; Sivanovic et al. |
| Line probe assay | 26.7 |  |
| 2nd line DST for 4 drugs (MGIT 960 liquid culture) | 37.83 |  |
| Full blood count | 9.2 |  |
| Liver function test (ALT, AST, Bilirubin) | 15.05 |  |
| Kidney function test (Potassium, Urea Creatinine) | 10.86 |  |
| Thyroid Stimulating Hormone | 20.89 |  |
| HIV rapid test | 5.26 |  |
| CD4 count and viral load | 51.75 |  |
| Pregnancy test | 2.89 |  |
| Chest X-ray | 31.91 |  |
| Audiogram | 25.6 |  |
| Smear | 6.3 |  |
| GeneXpert | 21.4 |  |
| ECG | 51.8 |  |
|  |  |  |
| **II. Standard MDR-TB drug regimen (per day)** |  | Eswatini Central Medical Stores |
| Drugs intensive phase | 7.1 |  |
| Drugs continuation phase | 6.3 |  |
|  |  |  |
| **III. Health system costs** |  | Sinanovic et al.; Cox et al.; study data |
| In-patient hospital stay (day) | 157 |  |
| Clinic visit (Out-patient consultation and collecting drugs) | 94 |  |
| Clinic visit (DOT and injection administration) | 24 |  |
| Home DOT and injection administration (visit) | 11 |  |
| DOT by family member | 4 |  |
| Supervision by community MDR-TB nurse (visit) | 19.4 |  |
| Financial support for patients intensive phase (per month) | 41.28 |  |
| Financial support for patients continuation phase (per month) | 8.26 |  |
| Food package support for patients (per month) | 33.03 |  |
| Financial incentives for CTSs (per month) | 57.8 |  |

MDR-TB – Multi-drug resistant tuberculosis, DOT – Directly Observed Treatment, MGIT – Mycobacterial Growth In-tube, DST – Drug susceptibility test, ALT - alanine aminotransferase, AST - aspartate aminotransferase; CTS: Community Treatment Supporter
