# Supplementary material for: Cost-consequence analysis of ambulatory clinic- and home-based multidrug-resistant tuberculosis management models in Eswatini
Source: PLoS One. 2024 Apr 2;19(4):e0301507. doi: 10.1371/journal.pone.0301507 (PMC10986922; doi:10.1371/journal.pone.0301507)
Supplement: S1 Appendix — (DOCX) [file pone.0301507.s002.docx]

**S1 Appendix: MDR-TB-related costs incurred during treatment**

**FOR OFFICIAL PURPOSES ONLY**

Data Collector: ____________________

Date Collected: ____________________

Health Facility: ____________________

| No. | *Umusho* | Statement | Amount (South African rands) |
| --- | --- | --- | --- |
| 1.16.1 | *Tindleko tekuvela emtfolamphilo* | Cost of consultation per visit |  |
| 1.16.2 | *Inombolo yekuvela emtfolapmhilo noma esibhedlela enyangeni leyengcile* | No. of consultations with the clinic/health centre in last month |  |
| 1.16.3 | *Tindleko tekutfola lusito lwetemphilo enyangeni leyengcile* | Total cost of consultation fees in last month |  |
| 1.16.4 | *Sikhatsi lesiphelele lesitsatfwa kuya nekubuya emtfolamphilo noma esibhedlela* | Total time taken (to and from) per visit to clinic/health centre |  |
| 1.16.5 | *Tindleko tekwekuhamba kuya emtfolamphilo noma esibhedlela* | Cost of transport per visit to the clinic/health centre |  |
| 1.16.6 | *Tindleko tekwekuhamba enyangeni leyengcile* | Total cost of transport in last month |  |
| 1.16.7 | *Tindleko tekuhlola kubapopoli enyangeni leyengcile* | Total cost of laboratory tests in past month |  |
| 1.16.8 | *Tindleko tekuhlola ku X-ray enyangeni leyengcile* | Total cost of X-rays in past month |  |
| 1.16.9 | *Tindleko letiphelele tekutsenga imitsi nemaphilisi enyangeni leyengcile* | Total cost of drugs purchased in past month |  |
| 1.16.10 | *Inombolo yemalanga ekungayi esibhedlela enyangeni leyengcile ngenca yekuya emtfolamphilo noma kulaliswa esibhedlela* | Total no. of days lost from work in last month (consultations and hospitalisation) |  |
| 1.16.11 | *Imali yemholo lephelele leyilahleke ngelilanga* | Total amount of wages lost per day |  |
| 1.16.12 | *Letinye tindleko letihambelana nekwelapha i MDR-TB enyangeni leyengcile.*  *Cacisa………………………………….* | Other costs associated with MDR-TB treatment in past month  Specify………………………………… |  |
|  | *Tindleko talonaka logulako* | Caregiver costs |  |
| 1.16.13 | *Umholo wenyanga* | Monthly income |  |
| 1.16.14 | *Tindleko tekwekuhamba uma yehluke naleyo yalogulako* | Transport cost if different from patient |  |
| 1.16.15 | *Inombolo yemalanga ekungaveli emsebentini kwenteela kupheketela logulako* | No. of days lost from work to accompany patient |  |
| 1.16.16 | *Imali yemholo lephelele leyilahleke ngelilanga* | Amount of wages lost per day |  |

***Siyabonga sikhatsi sakho usagcwalisa lelifomu***

**Thank you for taking time to complete this form**
