# Supplementary material for: Cost-consequence analysis of ambulatory clinic- and home-based multidrug-resistant tuberculosis management models in Eswatini
Source: PLoS One. 2024 Apr 2;19(4):e0301507. doi: 10.1371/journal.pone.0301507 (PMC10986922; doi:10.1371/journal.pone.0301507)
Supplement: S2 Appendix — (DOCX) [file pone.0301507.s003.docx]

**S2 Appendix: MDR-TB electronic medical records abstraction tool**

| MDR-TB registration number |  | |
| --- | --- | --- |
| **Medical information** | | |
| Diagnosis Date |  |  |
| Treatment outcome |  |  |
| Treatment outcome date |  |  |
| HIV co-morbidity | **Yes/No** |  |
| **Health system resources utilised** | | |
| **Element** | **Quantity** | |
|  | **Intensive Phase** | **Continuation Phase** |
| Doctor's consultation |  |  |
| Psychologist consultation |  |  |
| Counsellor |  |  |
| Admission (days) |  |  |
| Injection Admin Clinic/CTS |  |  |
| **Routine laboratory investigations** | | |
| Smear |  |  |
| GeneXpert |  |  |
| Culture + DST |  |  |
| Culture |  |  |
| CXR |  |  |
| ECG |  |  |
| Audiometry |  |  |
| Electrolytes |  |  |
| HbsAg |  |  |
| HTC |  |  |
| CD4 Count |  |  |
| Viral load |  |  |
| Pregnancy test |  |  |
| SGOT/SGPT |  |  |
| Chemistry |  |  |
| Creatinine |  |  |
| Urea |  |  |
| K+ |  |  |
| FBC |  |  |
| LFT |  |  |
| Uric acid |  |  |
| Total Bilirubin |  |  |
| TSH |  |  |
| CSF |  |  |
| Biopsy |  |  |
| CXR |  |  |
| Other |  |  |
| **MDR-TB drugs prescribed** | | |
| Pyrazinamide |  |  |
| Kanamycin 1 g vial |  |  |
| Capreomycin 1 g |  |  |
| Levofloxacin 250 mg |  |  |
| Moxifloxacin |  |  |
| Ethionamide 250 mg |  |  |
| Prothionamide |  |  |
| Cycloserine 250 mg |  |  |
| Teridizone |  |  |
| Ethambutol 400 mg |  |  |
| PAS |  |  |
| Clofazimine |  |  |
| Amoxy-Clav |  |  |
| Bedaquiline |  |  |
| Linezolide |  |  |
| High dose INH |  |  |
| Pyridoxine |  |  |
| CTX/Dapsone |  |  |
| TDF/3TC/EFV |  |  |
| TDF/3TC/NVP |  |  |
| AZT/3TC |  |  |
| EFV |  |  |
| 3TC |  |  |
| LPV/r |  |  |
| Other ARVs |  |  |
| **Other drugs prescribed** | | |
| Folate |  |  |
| FeSO4 |  |  |
| ART |  |  |
| Ondansetron |  |  |
| Metoclopromide |  |  |
| Plumby nut |  |  |
| Slow K |  |  |
| Mg Gluconate |  |  |
| Levothyroxine |  |  |
| Diclofenac gel |  |  |
| Diclofenac |  |  |
| Fluconazole |  |  |
| VTs |  |  |
| Paracetamol |  |  |
| Brufen |  |  |
| AMT |  |  |
