# Supplementary material for: Cost-consequence analysis of ambulatory clinic- and home-based multidrug-resistant tuberculosis management models in Eswatini
Source: PLoS One. 2024 Apr 2;19(4):e0301507. doi: 10.1371/journal.pone.0301507 (PMC10986922; doi:10.1371/journal.pone.0301507)
Supplement: Attachment — Submitted filename: Response to Reviewers.docx [file pone.0301507.s006.docx]

Centre for Development Support

Faculty of Economic and Management Sciences

University of the Free State

P.O. Box 399

Bloemfontein 9300

South Africa

Academic Editor

PLOS ONE

29 January 2024

**REF: Revision and resubmission of manuscript PONE-D-22-05265] - [EMID:5a0952a3e18fc9a6]**

Dear Editor

Thank you for your email and the opportunity to revise our paper on ‘Cost-consequence analysis of ambulatory clinic- and home-based multidrug-resistant tuberculosis management models in Eswatini.’ The suggestions offered by the reviewers have been immensely helpful in improving some important aspects of the manuscript.

We have included the academic editor and reviewers’ comments (in italics) immediately after this letter and responded to them individually, indicating exactly how we addressed each comment and describing the changes we have made. The revisions have been approved by all authors. The changes in the revised manuscript accompanying this letter are marked up and all page numbers refer to the revised manuscript file with tracked changes.

We hope the revised manuscript will better suit PLOS ONE, but will be happy to consider further revisions and we thank you for your continued interest in our research.

Sincerely,

Ernest Peresu

**Reviewer Comments and Author Responses**

**Reviewer 1**

***General comment:*** *Thank you for addressing my comments in your revision - a lot of hard work went into this work.*

**Response:** We would like to thank the reviewer for carefully and thoroughly reading our manuscript and for the thoughtful and encouraging comments. We have incorporated these insightful suggestions to improve our paper.

***Comment 1:*** *For the sentence starting “The higher risk of unfavourable outcomes among those patients delayed initiating MDR-TB treatment has been consistently reported in studies from elsewhere.”* *Please indicate two to three examples of unfavourable outcomes associated with delayed treatment initiation of MDR-TB.*

**Response:** Thank you for pointing this out. We found this comment very helpful. We have added two unfavourable outcomes associated with delayed initiation of MDR-TB treatment (line 402 – 405). The sentence now reads:

*“The elevated risk of unfavourable outcomes such as high mortality and increased person-to-person transmissions among those patients who delayed initiating MDR-TB treatment has been consistently reported in studies from elsewhere.”*

***Comment 2:*** *Table 3: rename the table title as “summary of key treatment management indicators for MDR-TB patients” and report (for each component and for each intervention) sample size used to calculate each summary statistics. Also, summary statistics can either be median or mean (based on the distribution of the data) and the authors should report uncertainty ranges (95% CI or IQR).*

**Response:** We appreciate the reviewer’s insightful suggestion and we have renamed Table 3 (line 283 – 284). We have also modified and improved Table 3 by presenting the mean and the associated 95% confidence interval for each treatment component variable for the respective strategy.

***Comment 3:*** *Line 515-521: How does no including cost for laboratory investigation in this study relates to Pooran et al.,’s finding? Since this is a cost consequence analysis, not including certain key health care cost estimates essentially under-estimates actual cost (for each arm) of MDR-TB care. In cost-effectiveness analysis, these costs may cancel out in calculating ICER, but in this analysis, providing an overall cost estimate is important as the authors are not calculating ICER. Authors should state that the limitations in not including key health care costs for MDR-TB care, especially on diagnostics, under-estimates the overall MDR-TB care costs. If authors can provide specific items that are not included, they can suggest by how much (approximately) the authors MDR-TB care estimates is under-estimated.*

**Response:** This is an excellent comment, and we agree that diagnostic costs are any important component that should be included in a cost-consequence analysis assessment study. In this study, we indeed considered and reported the cost of laboratory diagnostic and monitoring tests in supplementary S1 Table (line 189 – 190); in text (line 293 – 297) and in Table 4 (line 299 – 302). The total laboratory test costs were determined by multiplying unit costs by the frequency of tests performed for the period of MDR-TB treatment as recommended in the national TB policy guidelines. Given that the unit cost of each component associated with diagnosis of MDR-TB in Eswatini was not readily available from the Eswatini National TB Control Programme (NTCP), our study used unit cost data reported by Pooran and colleagues in a study conducted in South Africa titled *“What is the Cost of Diagnosis and Management of Drug Resistant Tuberculosis in South Africa?”.* There is negligible variation in MDR-TB clinical practices, life expectancy, payment systems and discount rate between Eswatini and South Africa – in a way enabling the geographic transferability of unit cost data for each laboratory diagnostic component.

***Comment 4:*** *Line 526-528: Strength comment as a separate paragraph may not be necessary as it’s being discussed in the earlier section. Please delete. Line 484-486.*

**Response:** This is a great observation and indeed true. We have deleted the strength comment (line 486 – 488).

***Comment 5:*** *Line 532: delete “although qualified by the study limitations” (not necessary). Line 489.*

**Response:** Thank you for this suggestion. We have deleted *“although qualified by the study limitations”* (line 492).
